# Porous (PVDF-HFP/PANI/GO) ternary hybrid polymer electrolyte membranes for lithium-ion batteries

**DOI:** 10.1039/c8ra03918f

**Published:** 2018-07-18

**Authors:** A. L. Ahmad, U. R. Farooqui, N. A. Hamid

**Affiliations:** School of Chemical Engineering, Universiti Sains Malaysia, Engineering Campus 14300 Nibong Tebal Pulau Pinang Malaysia chlatif@usm.my urf222@gmail.com chrina@usm.my

## Abstract

A poly(vinylidene *co*-hexafluoropropylene) (PVDF-HFP) membrane is functionalized with polyaniline (PANI) and graphene oxide (GO) nanoparticles. The obtained PVDF-HFP polymer electrolyte membranes (PEMs) have been characterized and implemented in lithium-ion batteries. As a result, the PVDF-HFP/PANI membrane shows the highest ionic conductivity (IC) of 1.04 × 10^−3^ mS cm^−1^ compared to pristine PVDF-HFP and PVDF-HFP/PANI/GO ternary membrane; however, PANI addition decreases the tensile strength of the PVDF-HFP membrane from 4.2 MPa to 2.8 MPa. Therefore, GO is introduced to recover the reduced mechanical strength of the PVDF-HFP/PANI membrane. The obtained PVDF-HFP/PANI/GO ternary membrane shows a remarkable improvement in tensile strength of up to 8.8 MPa; however, slight reduction is observed in the ionic conductivity of 6.64 × 10^−4^ mS cm^−1^. Furthermore, the PVDF-HFP/PANI/GO ternary membrane exhibits outstanding thermal and mechanical stabilities, improved morphology, highest electrolyte uptake (367.5%) and an excellent porosity of around 89%. Moreover, the PVDF-HFP/PANI/GO ternary PEM has been successfully applied in a lithium-ion battery, which can retain over 95% capacity after 30 cycles. Therefore, the proposed PVDF-HFP/PANI/GO ternary membrane can be a promising candidate as a separator in future lithium-ion batteries.

## Abstract 2

A poly(vinylidene co-hexafluoropropylene) (PVDF-HFP)/polyaniline (PANI/graphene oxide (GO) ternary PEM in lithium ion battery.

## Graphical abstract

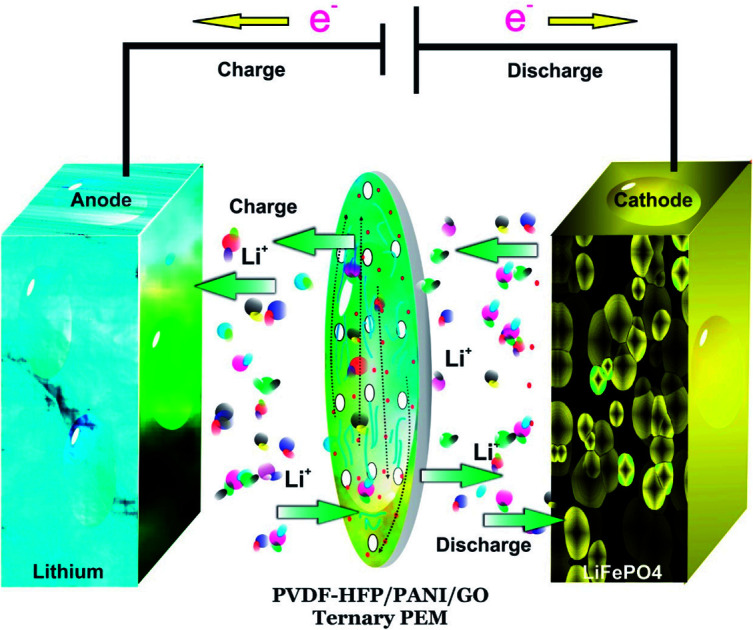

## 1 Introduction

Lithium-ion batteries (LIBs) have shown remarkable potentials as energy storage devices compared to various other possible alternatives.^1^ The high efficiency and excellent energy density of lithium-ion batteries make them a promising power source for hybrid-electronic vehicles, electric vehicles, and portable electronic devices; also, they can be used to store electrical energy from renewable sources such as wind or sun.^2,3^ However, LIBs still need extensive research in terms of safety and performance optimization. The concepts of liquid electrolytes and solid polymer electrolytes have been recommended as solutions to some of the issues related to LIBs;^4–7^ however, the low ionic conductivities of solid polymer electrolytes and the leakage issues associated with liquid electrolytes limit their performances in LIBs.^8^

Therefore, polymer electrolyte membranes or gelled polymer electrolyte membranes (PEMs) have received tremendous attention in the last few years. PEMs have shown better performances compared to their other counterparts such as solid electrolyte membranes and liquid electrolyte membranes. The use of PEMs promotes safe, lightweight and leakage proof construction of lithium batteries with improved capacity and cycle life.^9^ Nowadays, the focus is more on producing PEMs with enhanced room-temperature conductivity, better ion transportation and stable interfacial properties of electrolytes and electrodes.^10–12^ In this regard, several polymers such as poly(methyl methacrylate),^8^ poly(vinylidene fluoride) (PVDF),^13^ polyacrylonitrile (PAN)^12,14^ and poly(vinylidene fluoride-*co*-hexafluoropropylene) (PVDF-HFP)^15,16^ have shown good performances when applied in lithium batteries; however, these polymers have shown limited characteristics in their pristine form. Therefore, the functionalization of host polymers either with fillers or additives or by blending pure polymer matrix with other polymers has a significant role in performance optimization. In this respect, various fillers such as ZrO_2_, TiO_2_, SiO_2_, graphene oxide (GO), polyaniline (PANI) and Al_2_O_3_ have been used to functionalize the pure polymer matrix;^17–23^ their addition has resulted in improved mechanical strength and reduced crystallinity, which ultimately improves the ionic transport and enhances the ionic conductivity of PEMs.^4,17,24–27^ Among several polymers, PVDF-HFP has shown good performance and flexibility for further modifications similar to PEMs; it has amorphous HFP and a crystal VDF, which provide better ionic conductivity and improved mechanical stability to the membrane.^7,28–30^

Therefore, a novel PVDF-HFP/PANI/GO ternary hybrid membrane has been prepared, characterized and applied in lithium-ion batteries. Polyaniline has shown great impact on energy devices due to its flexible conductive nature, easy synthesis and better interaction with host polymers and fillers;^31^ however, it has rarely been tested and verified as a separator in a lithium battery. Subsequently, GO is widely used in energy-related applications since its introduction in the last few years; it has shown great interaction with PANI due to its oxygenated functional groups.^32,33^ Even though the PANI/GO composite has been reported in several studies, it has never been tested as a PEM or as a separator in a lithium-ion battery. Both PANI and GO have unique properties, and it will be interesting to investigate the effect of the PANI/GO composite on the performance of the PVDF-HFP separator in an LIB. Thus, the proposed PVDF-HFP/PANI/GO ternary hybrid PEM can be a promising alternative to separators in future lithium-ion batteries.

## 2 Materials and methods

### 2.1 Materials

Aniline (99%), graphene oxide powder, ammonium persulfate (99.99%), ammonium hydroxide (28%), hydrochloric acid (37%, reagent grade), *n*-butanol, poly (vinylidene *co*-hexafluoropropylene) (PVDF-HFP) pellets (99.99%), acetone (99.9%), ethanol and NMP were purchased from Sigma Aldrich.

### 2.2 Methods

**Fig. 1:**
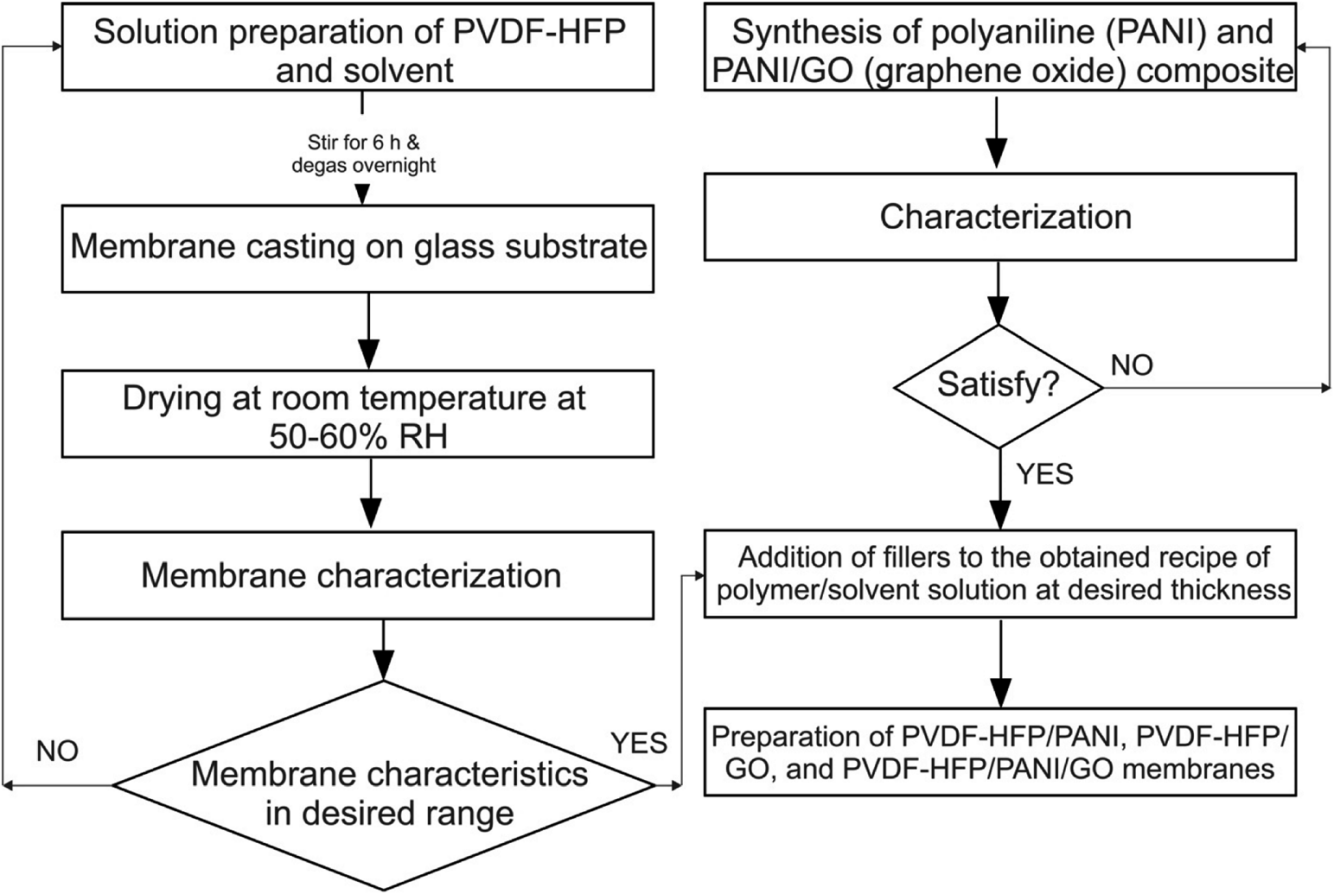
Flow chart of the membrane preparation.

The flow chart of the whole methodology is shown in Fig. 1.

#### 2.2.1 Synthesis of polyaniline (PANI) and PANI/GO composite material

The simple conventional polymerization method was used to synthesize PANI/GO composite nanoparticles.^34^ Briefly, PANI synthesis involves the addition of 10% aniline monomer to 1 M HCl solution. After that, the oxidizing agent 0.1 M ammonium persulfate (APS) was added dropwise to the solution to initiate polymerization. After continuous stirring of the solution in an ice bath for around 2 h, the solution was kept in a refrigerator overnight. After that, the obtained green colored solution was filtered and washed with ethanol and water to remove its impurities. Subsequently, the dark-green colored residue was treated with ammonium hydroxide (NH_4_OH) for around 24 h until its color changed to blue; then, it was washed by ethanol and water before its further use.

#### 2.2.2 Synthesis of PVDF-HFP and PVDF-HFP/PANI/GO ternary membranes

The pure and modified PVDF-HFP polymer membranes were prepared through the breath figure method.^35^ In brief, PVDF-HFP (15 wt%) pellets were dissolved in a mixture of acetone and NMP solvent with a 40 : 60 ratio and allowed to stir for 24 h; then, the mixture was kept at room temperature throughout the night for bubble removal. After that, the solution was cast on a glass substrate and allowed to dry at room temperature with around 45–60% RH. After complete solvent evaporation, the membranes were peeled off and stored in an argon glove box.

Likewise, PVDF-HFP/PANI (PANI = 2 wt%) and PVDF-HFP/PANI/GO [PANI : GO (60 : 40)] ternary hybrid membranes were prepared by the breath figure method. For PEMs, the prepared membrane samples were dipped in 1 M EC : DMC [1 : 1] lithium ion phosphate (LiPF_6_) solution for about 24 h before further testing.

### 2.3 Physical and electrochemical characterization

Scanning electron microscopy (SEM) analysis was performed with a FESEM, Zeiss Supra 35VP instrument to observe the morphology of the membrane. The thermal stability of different membrane samples was determined by thermogravimetric (TGA) analysis; TGA and differential scanning calorimetry (DSC) analyses were performed at 10 °C min^−1^ from 0 to 400 °C with Perkin Elmer STA-6000 and DSC-4000, respectively. Furthermore, Fourier transform infrared spectroscopy (FTIR) analysis was conducted within the range of 500–4000 cm^−1^ with a Nicolet iS10 spectrometer by Thermoscientific. Subsequently, X-ray diffraction (XRD) analysis was performed to detect the phase of the host polymer from 10 to 90° using SIEMEN XRD (D5000). Moreover, the mechanical stability tests of different membrane samples were performed at 10 kN as per ASTM D882-10 standards by using an Instron 3366 instrument; membrane samples with a length of 10 cm and a width of 1.5 cm were tested at a speed of 2 mm min^−1^ through series IX software.

Different membrane samples were cut into 2 cm × 2 cm size and dipped into LiPF_6_ electrolyte and *n*-butanol for 2 h to measure electrolyte uptake and porosity, respectively. The excess liquid was wiped off from the surface, and the weights were measured before and after immersion of the membrane samples. The following equations were used to determine electrolyte uptake and porosity:Porosity = (*W*_f_ − *W*_i_)/(*V*_dry_ × *ρ*_b_)Electrolyte uptake = (*M*_f_ − *M*_i_) × 100/*M*_i_here, *M*_i_, *M*_f_, and *W*_i_, *W*_f_ represent the weights of the membrane samples before and after immersion in liquid electrolyte and *n*-butanol, respectively; *ρ*_b_ is the density of *n*-butanol and *V*_dry_ is the volume of the dry membrane samples.

The ionic conductivities of PEM samples were measured by sandwiching them between two stainless steel electrodes. Electrochemical impedance spectroscopy (EIS) analysis was performed by using a VMP3 battery analyzer and the EC-lab software at room temperature. The bulk resistances were obtained through EIS complex graphs, which were used to determine the ionic conductivity by the following relation:*σ* = *d*/(*R*_b_ × *S*)here, *d* is the thickness and *S* is the surface area of different membrane samples. Chronoamperometry (CA) analysis was performed with the help of a VMP3 battery analyzer at 5 V for 30 min to determine the lithium ion transference number by using the following equation:*T*_Li^+^_ = *I*_s_/*I*_o_here, *I*_s_ and *I*_o_ represent the currents at steady state and initial state, respectively.

The coin cell CR2032 was prepared to evaluate the performance of the lithium-ion battery. The prepared membrane samples were used as separators, whereas lithium metal and LiFePO_4_ carbon were used as the anode and cathode, respectively. For the working electrode preparation, a mixture of Katzen black (10 wt%), PVDF binder (10 wt%) and LiFePO_4_ (80 wt%) was dissolved in *N*-methyl-2-pyrrolidone (NMP) solvent and pasted on a stainless steel grid. Subsequently, the mixture was vacuum dried at 80–90 °C for around 12 h before its further use. The assembly was stored in an argon glove box with oxygen and moisture concentrations of less than 0.1 ppm.

## 3 Results and discussion

**Fig. 2:**
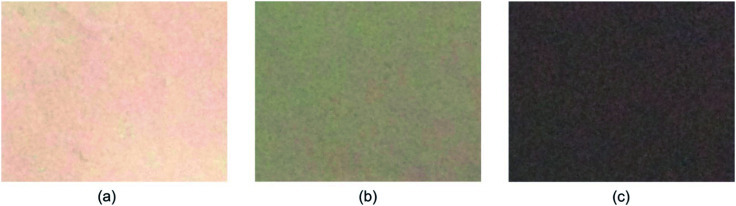
Real images of (a) pristine PVDF-HFP, (b) PVDF-HFP/PANI, (c) PVDF-HFP/PANI/GO PEMs.

**Fig. 3:**
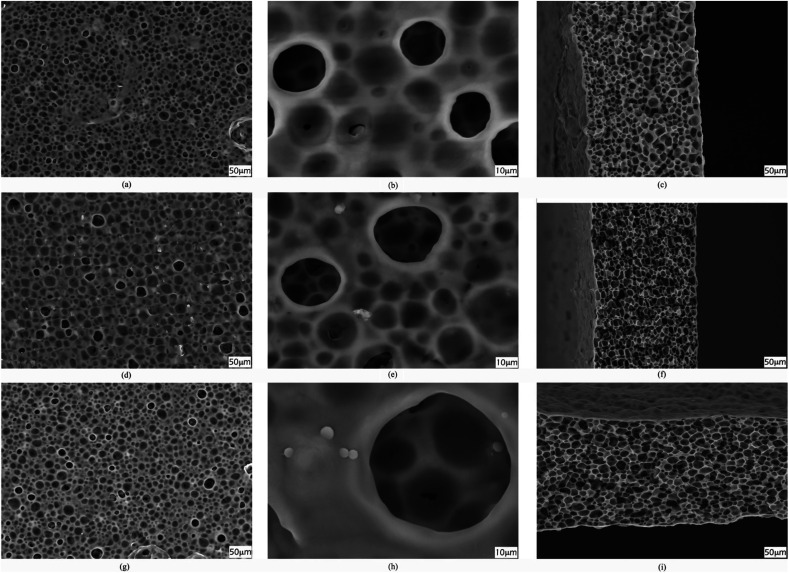
SEM images of surfaces (a and b), (d and e) and (g and h); cross sections (c), (f) and (i) of pristine PVDF-HFP, PVDF-HFP/PANI and PVDF-HFP/PANI/GO membranes, respectively.

The procedure for membrane preparation has been reported in the above-mentioned section. The real images of various prepared membranes are shown in Fig. 2. The thickness of the prepared PVDF-HFP membranes is around 65 ± 5 μm. As shown in Fig. 3, a significant difference can be observed in the surfaces and cross-sectional morphologies of different PVDF-HFP membranes. The GO addition results in an excellent uniform porous structure of the PVDF-HFP/PANI/GO ternary membrane as compared to those of pure and PVDF-HFP/PANI membranes; also, its incorporation in the ternary membrane remarkably improves the porosity from around 68% to 89% and EU from about 296% to 367.5% when compared to the observations for pristine PVDF-HFP membrane. Furthermore, the filler alteration to the PVDF-HFP polymer introduces some smaller pores under the larger pores of the membrane; the smaller pores can avoid dendrite formation in batteries, and larger pores can increase the electrolyte uptake by holding the electrolyte more effectively. In addition, the improved morphology of the ternary membrane is also ascribed to the breath figure method and the unique solvent mixture of NMP and acetone. NMP is an excellent solvent for both PANI and GO dispersion;^31,36^ hence, it facilitates the interaction between fillers and improves their dispersion in the host polymer. Acetone evaporates quickly due to its lower boiling point and provides initial stability to the membrane straight after casting; also, the breath figure method favors water droplet formation on the membrane surface, which further enhances the morphology of the membrane. Overall, the proposed modified breath figure method and the addition of the PANI/GO composite to the host polymer result in an excellent PVDF-HFP/PANI/GO ternary hybrid membrane.

**Fig. 4:**
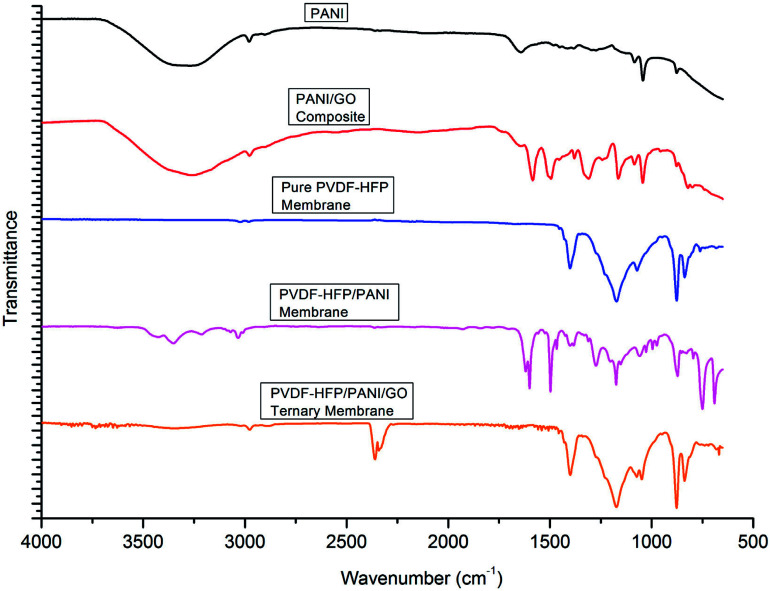
FTIR analysis of PANI, PANI/GO composite, pristine PVDF-HFP, PVDF-HFP/PANI and PVDF-HFP/PANI/GO ternary membranes.

As shown in Fig. 4, FTIR analysis was performed for different particles and membranes to investigate the interactions among them. The characteristic peaks originated at 1380 cm^−1^ (benzenoid (B) ring stretching), 1644 cm^−1^ (quinonoid (Q) ring stretching), 1272 cm^−1^ (C–N stretching), 876 cm^−1^ (aromatic C–H vibration) and 1083 cm^−1^ (N=Q=N stretching) confirmed the presence of PANI particles. Likewise, the peaks obtained at 1185 cm^−1^ (C–O stretching), 1714 cm^−1^ (C=O stretching), and 3852 cm^−1^ (O–H stretching) were assigned to GO particles. The PANI/GO composite spectrum showed characteristic peaks at 1160 cm^−1^ (C–H deformation), 1582 cm^−1^ (quinoid vibrations), 1377 cm^−1^ (absorption band) and 3260 cm^−1^ (O–H stretching). Also, the peak obtained at 1382 cm^−1^ (benzenoid) for PANI slightly shifted to 1308 cm^−1^ for the PANI/GO composite possibly due to the interaction of H-bonding and π–π bonding between PANI and GO structure. Subsequently, different spectra were observed for different PVDF-HFP PEMs. The characteristic peaks at 835 cm^−1^ (CF3 stretching), 870–880 cm^−1^ (vinylidene group), 1170 cm^−1^ (CF2 group) and 1398 cm^−1^ (CH=CF skeleton) affirmed the existence of the PVDF-HFP polymer. In another spectrum, a few different peaks such as 790–800 cm^−1^ (phenyl group), 1173 cm^−1^ (C–N stretching), 1615 cm^−1^ (C=C stretching) and 3278 cm^−1^ (N–H stretching) were observed for the PVDF-HFP/PANI membrane. Furthermore, a significant difference could be seen in the spectra of the PVDF-HFP/PANI/GO ternary membrane. The peaks obtained at 839 cm^−1^ (CF3 stretching), 1050 cm^−1^ (C–C skeleton), 1400 cm^−1^ (C=N stretching), 2300–2500 cm^−1^ (aromatic C–H stretching), 3852 cm^−1^ (O–H stretching), and 3332 cm^−1^ (N–H stretching) all suggested the successful incorporation of the PANI/GO composite into the PVDF-HFP polymer matrix.^37–39^

**Fig. 5:**
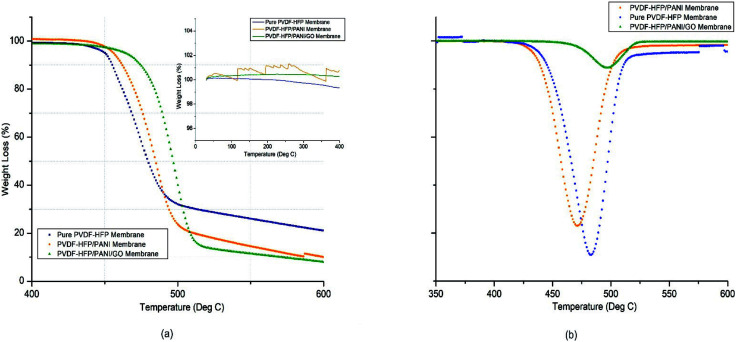
Thermogravimetric (TGA) analysis of different PVDF-HFP membranes.

PEMs must have good thermal stability to perform well in energy devices. Thus, TGA analysis was used to analyze the thermal stability of different membranes. As shown in Fig. 5 and reported in Table 1, there was remarkable improvement from pure to nanoparticle-modified PVDF-HFP PEMs. The PVDF-HFP/PANI/GO ternary membrane had the highest thermal degradation temperature (*T*_d_) at around 498 °C possibly due to the effective interaction of the PANI/GO composite with the PVDF-HFP polymer matrix, and the *T*_d_ values were about 470 °C and 484 °C for pristine PVDF-HFP and PVDF-HFP/PANI membranes, respectively. Furthermore, only around 14% mass loss was noticed for the PVDF-HFP/PANI/GO membrane between 440 °C and 480 °C, whereas the values were about 49% and 31% for pristine PVDF-HFP and PVDF-HFP/PANI membranes, respectively. As shown in the inner graph of Fig. 3(a), the PVDF-HFP/PANI/GO ternary membrane was found to be the most stable up to 400 °C compared to others; however, the pristine PVDF-HFP and PVDF-HFP/PANI membranes also showed good stabilities with only slight phase degradations at 250 °C and 200 °C, respectively.

**Table 1:** *T*_m_, *T*_d_, degree of crystallization (*X*_c_), EU, porosity and ionic conductivity of different PVDF-HFP membrane samples

Sr. no.	Membrane sample	*T*_d_ (°C)	*T*_m_ (°C)	% *X*_c_	Electrolyte uptake (%)	Porosity (%)	Ionic conductivity (mS cm^−1^)
1	Pure PVDF-HFP membrane	470.34	138.37	69.2	296.4	68.8	1.98 × 10^−4^
2	PVDF-HFP/PANI membrane	483.34	129.95	48.8	340.4	77.3	1.04 × 10^−3^
3	PVDF-HFP/PANI/GO ternary membrane	497.50	125.95	51.9	367.6	88.7	6.64 × 10^−4^

**Fig. 6:**
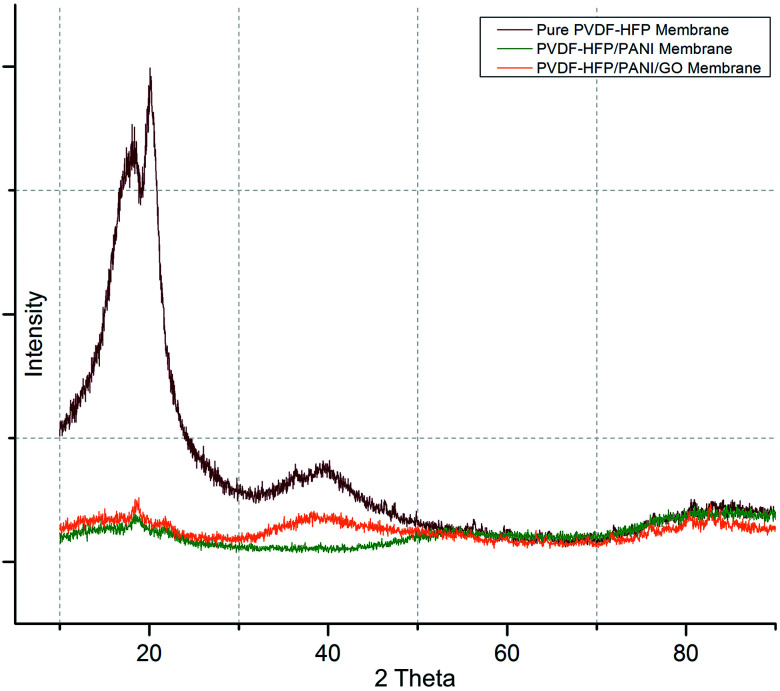
XRD analysis of various PVDF-HFP membrane samples.

Subsequently, DSC and XRD analyses were performed to investigate the effects of nanoparticle addition on the semi-crystalline nature of the PVDF-HFP membrane. As shown in Fig. 6, first, XRD analysis affirmed the semi-crystalline nature of the PVDF-HFP membrane with characteristic peaks observed at 2 theta = 18.4, 20, and 36. Interestingly, the highest crystallinity reduction was found for the PVDF-HFP/PANI membrane; however, the PVDF-HFP/PANI/GO ternary membrane showed better reduction as compared to the pristine PVDF-HFP membrane. Both the GO and PANI fillers are amorphous in nature; hence, both showed reduced peak intensities compared to the pristine PVDF-HFP membrane. Also, their combination, *i.e.*, the PANI/GO composite material, produced almost similar results to those obtained with the PVDF-HFP/PANI membrane; however, PANI alone was found to be the most effective in terms of crystallinity reduction in PVDF-HFP PEM. Additionally, higher amorphous region facilitated lithium ion transport through the membrane, which eventually enhanced the ionic conductivity. Therefore, the filler-modified membranes have more potential to perform compared to the pristine PVDF-HFP membrane in lithium-ion batteries.

**Fig. 7:**
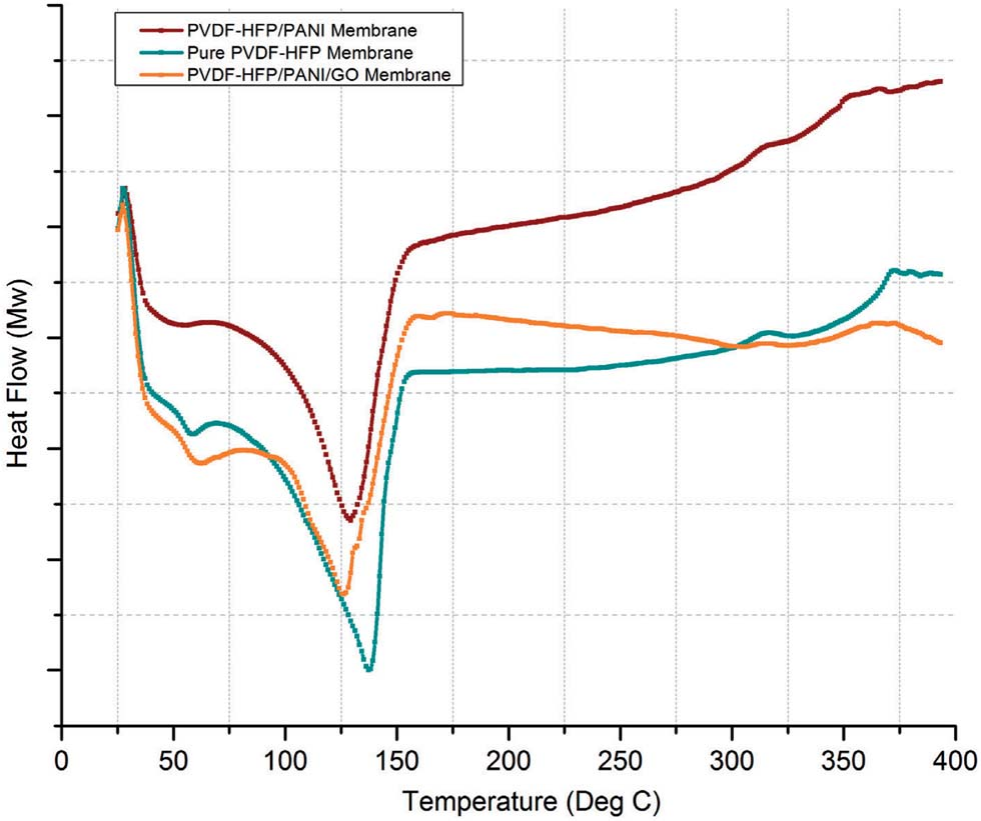
DSC analysis of different PVDF-HFP membrane samples.

Similarly, DSC analysis has been performed to cross-check the XRD investigations and also to obtain the melting temperatures (*T*_m_) of various membranes. As shown in Fig. 7, a significant difference in *T*_m_ can be seen for pure PVDF-HFP and modified PVDF-HFP membranes. The broader endothermic peak shows a *T*_m_ value that is highest for the pure PVDF-HFP membrane at around 139 °C; however, it shifts towards the left and results in reduced *T*_m_ values for the modified PVDF-HFP membranes. The lowest *T*_m_ value is obtained at around 125 °C for the PVDF-HFP/PANI/GO ternary membrane as the PANI/GO addition disturbs the crystal structure of the PVDF-HFP polymer. However, the PVDF-HFP/PANI ternary membrane also shows good reduction in *T*_m_ with a value of about 130 °C due to PANI nanoparticles, which can disrupt the crystal structure of the PVDF-HFP membrane. Moreover, the crystallization degree can be calculated through the area under the *T*_m_ curve by the equation given below:*X*_c_ = Δ*H*_m_/Δ*H*_m°_here, Δ*H*_m_ and Δ*H*_m°_ represent the fusion enthalpy of the prepared PEMs and that of the pure PVDF-HFP polymer (*i.e.*, 104.7 J g^−1^), respectively.

**Fig. 8:**
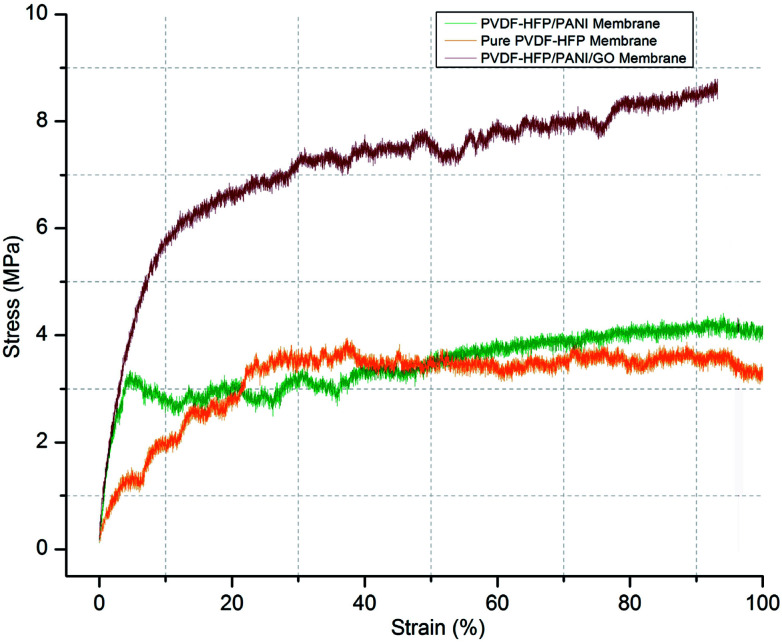
Tensile strength of different PVDF-HFP membranes.

In addition, the mechanical stability of modified and unmodified PVDF-HFP PEMs is a major concern for the separator in lithium-ion batteries. In this regard, the tensile strength test of different PVDF-HFP membranes has been performed at room temperature. As shown in Fig. 8, the PVDF-HFP/PANI/GO ternary membrane with 8.9 MPa displays much better tensile strength when compared to the pure PVDF-HFP membrane (4.2 MPa). In contrast, the tensile strength of the PVDF-HFP/PANI membrane reduces to 2.8 MPa, which can be due to the plasticizing effect of PANI; this decreases the flexibility and breaks it slightly earlier than that observed in other PVDF-HFP membranes. However, the PANI/GO composite responds well in the PVDF-HFP polymer matrix and forms a balanced ternary membrane. The improved tensile strength of the ternary membrane is due to the enhanced interfacial contact area between the polymer chains of PVDF-HFP and the GO particles. The polymer chains of the host polymer enwrap the GO particles and enhance the mechanical stability of the composite PEMs. Thus, GO addition to the PANI particles balances the reduced mechanical strength of PVDF-HFP/PANI PEM, which results in an improved PVDF-HFP/PANI/GO ternary hybrid PEM.

**Fig. 9:**
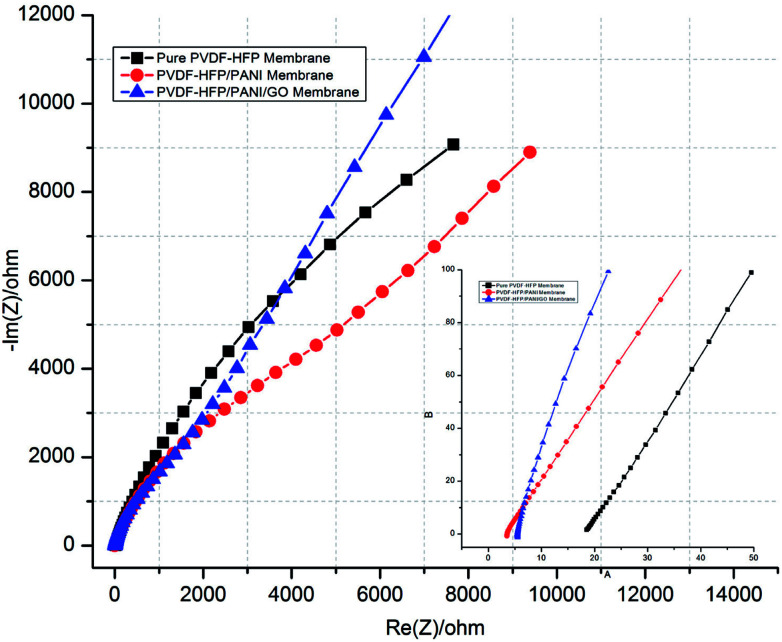
EIS complex graphs of various PVDF-HFP PEMs.

**Fig. 10:**
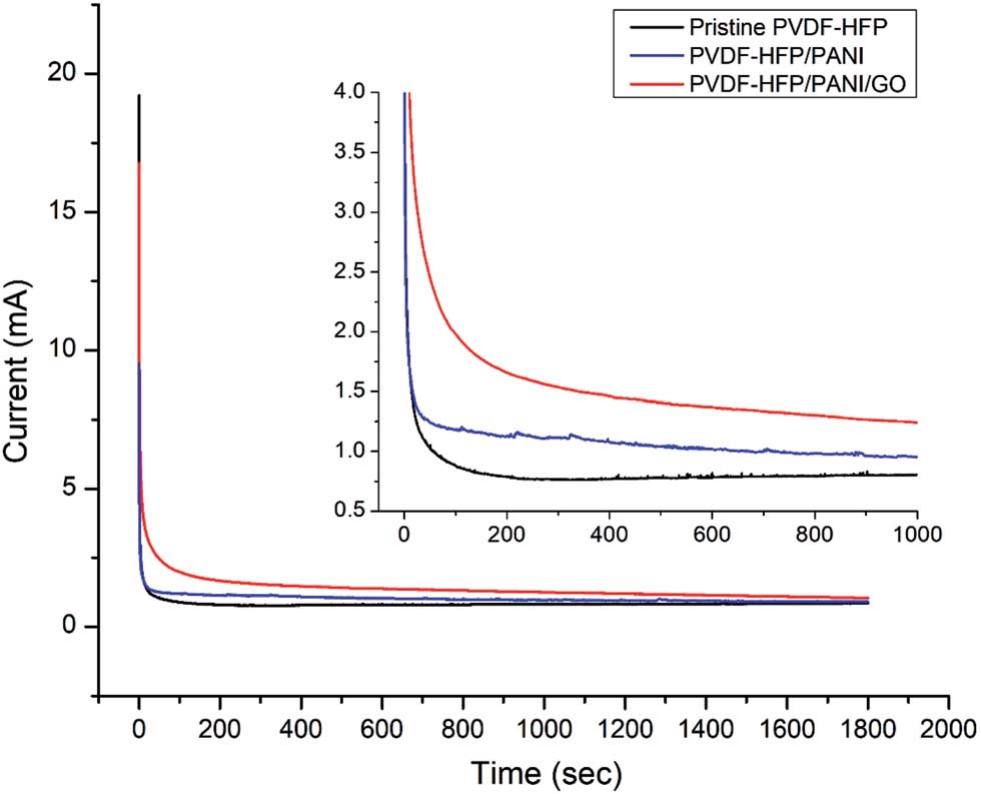
Chronoamperometry profiles of various PVDF-HFP membranes.

Moreover, ionic conductivity is also an important factor for the membrane separator in lithium batteries. As shown in Fig. 9 and reported in Table 1, the addition of PANI to the PVDF-HFP polymer matrix results in an excellent ionic conductivity of 1.04 × 10^−3^ mS cm^−1^ possibly due to the improved electrostatic force of attraction in the polymer matrix. In contrast, GO insertion is not very effective in terms of ionic conductivity and produces around 6.64 × 10^−4^ mS cm^−1^ with the PVDF-HFP/PANI/GO ternary membrane; nevertheless, this value is still better compared to those of pure and other reported PVDF-HFP membranes. Recently, an ionic conductivity of 4.23 × 10^−4^ mS cm^−1^ has been reported for the PVDF-HFP/GO membrane.^40^ Similarly, ionic conductivities of 4.98 × 10^−3^ mS cm^−1^,^41^ 0.918 × 10^−3^ mS cm^−1^ (ref. 42) and 1.31 × 10^−3^ mS cm^−1^ have been reported for PVDF-HFP/PVA/LiCF_3_SO_3_/LiAlO_2_, PVDF-HFP/PEMA and PVDF-HFP/PMMA membranes, respectively; membranes with ionic conductivities over 10^−3^ are acceptable for lithium batteries.^43^ Therefore, the PVDF-HFP/PANI/GO ternary hybrid membrane can be considered to be a balanced PEM as it exhibits better ionic conductivity as well as excellent mechanical strength compared to the pure PVDF-HFP membrane. Moreover, chronoamperometry analysis provides the lithium ion transference number (*T*_Li^+^_) for different PVDF-HFP membranes, as shown in Fig. 10. The highest *T*_Li^+^_ value of around 0.30 is obtained with the PVDF-HFP/PANI/GO ternary membrane, whereas *T*_Li^+^_ values of about 0.20 and 0.13 are obtained for PVDF-HFP/PANI and pristine PVDF-HFP membranes, respectively.

**Fig. 11:**
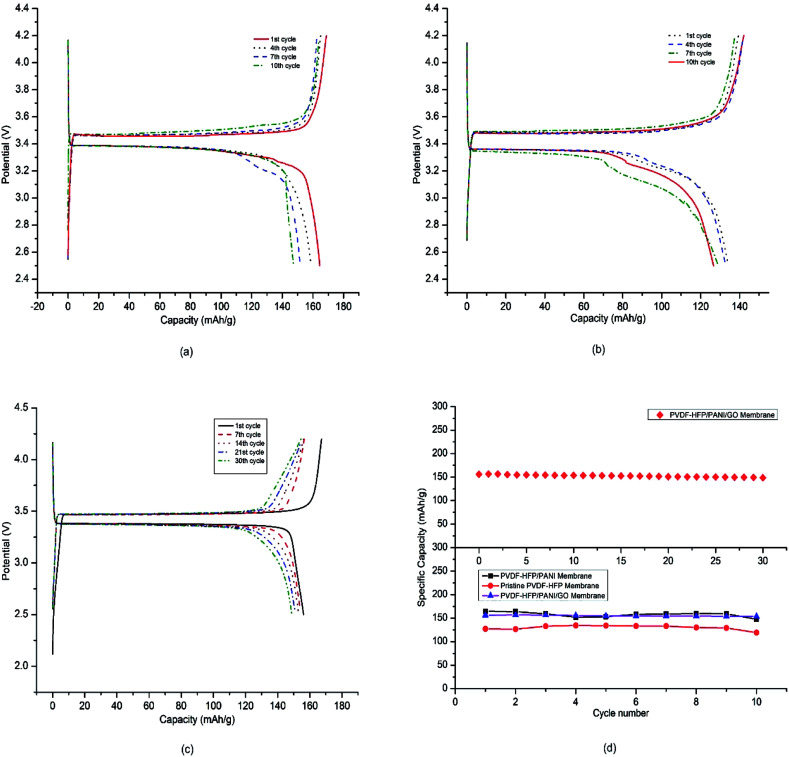
Electrochemical performances of lithium ion coin cells: (a)–(c) charge–discharge curves for PVDF-HFP/PANI, pristine PVDF-HFP and PVDF-HFP/PANI/GO PEM, respectively; (d) cycling performance of various PVDF-HFP PEMs at cut off voltages of 2.5–4.2 V.

Furthermore, the coin cell CR2032 was used to test the electrochemical performances of different PEMs in lithium-ion cells. The prepared membranes were used as separators, lithium metal was used as the anode, and LiFePO_4_ was used as the cathode in the coin cell assembly. The battery coin cells were tested at a C/20 current rate with a cut-off voltage of 2.5–4.2 V. As shown in Fig. 11(a)–(c), the charge–discharge curves of various lithium-ion coin cells with different PVDF-HFP membrane separators exhibited plateaus at around 3.4 V. In addition, the voltage difference between the charge–discharge curves was not more than 0.1 V, which can normally be obtained with commercial separators when used directly with the electrolyte.^44,45^ A very low voltage difference between the charge–discharge curves for around 30 cycles demonstrated the capability of the PVDF-HFP/PANI/GO ternary membrane as an important energy device. Fig. 11(d) describes the obtained initial discharge capacities of pristine PVDF-HFP, PVDF-HFP/PANI, and the PVDF-HFP/PANI/GO ternary hybrid membranes, which were around 127 mA h g^−1^, 164 mA h g^−1^ and 156 mA h g^−1^, respectively; however, the PVDF-HFP/PANI/GO ternary membrane was found to be more stable during the initial 10 cycles compared to the others. Even though the cell discharge capacity of LIBs is highly dependent on the cathode material, the morphology, ionic conductivity and electrolyte uptake of the membrane separator also have significant effects on it. Therefore, based on the overall performance, the PVDF-HFP/PANI/GO ternary membrane was subjected to the capacity retention test, and excellent results were obtained with more than 95% capacity retention after 30 cycles; this showed that with the ternary membrane, the battery could easily go up to 30 cycles without significant degradation in capacity. The electrode materials could remain wet more effectively for a longer time period due to larger electrolyte uptake, which ultimately enhanced the ionic conductivity and the cell performance. Thus, the performances of all the tested membranes were satisfactory for the initial 10 cycles; however, the addition of the PANI/GO composite and its effective interaction with the host polymer resulted in a very balanced PVDF-HFP/PANI/GO ternary hybrid membrane. The improved ionic conductivity of the PVDF-HFP membrane was due to the addition of PANI, whereas the excellent mechanical stability was due to GO incorporation. Moreover, the PANI/GO composite material showed the best morphology, which enhanced the porosity and electrolyte uptake of the PVDF-HFP/PANI/GO ternary hybrid membrane. Therefore, a very stable and efficient PVDF-HFP/PANI/GO ternary hybrid membrane was obtained, and it showed great potential as a separator in lithium-ion batteries.

## 4 Conclusion

A PVDF-HFP/PANI/GO ternary hybrid membrane was successfully fabricated by the breath figure method. The obtained ternary membrane was compared to the pristine PVDF-HFP membrane and the PVDF-HFP/PANI membrane in terms of various physical and electrochemical properties. The PVDF-HFP/PANI membrane showed the highest ionic conductivity with a value of 1.04 × 10^−3^ mS cm^−1^; however, decreased tensile strength was observed from 4.2 MPa of the pure PVDF-HFP membrane to 2.8 MPa due to its plasticizing effect. Therefore, GO addition resulted in the highest tensile strength of 8.9 MPa for the PVDF-HFP/PANI/GO ternary hybrid membrane; however, GO addition exhibited negligible effect on the ionic conductivity of the PVDF-HFP/PANI membrane. Thus, the unique combination of PANI/GO composite material efficiently balanced the ionic conductivity and the mechanical strength of the PVDF-HFP polymer matrix. The new and improved PVDF-HFP/PANI/GO ternary hybrid membrane showed excellent morphology with the highest porosity of around 89% and the highest electrolyte uptake of about 367.5%. Moreover, all the PEMs were successfully tested with a battery cycler through the coin cell CR2032. Every membrane displayed good rate performance when implemented in lithium-ion batteries; also, the proposed PVDF-HFP/PANI/GO ternary membrane displayed excellent rate performance and retained over 95% of the cell capacity after 30 cycles. Therefore, the proposed PVDF-HFP/PANI/GO ternary membrane can be a promising alternative separator for lithium-ion batteries.

## Conflicts of interest

There are no conflicts of interest.

## Supplementary Material

## References

[cit1] Manuel Stephan A., Nahm K. S. (2006). Polymer.

[cit2] Scrosati B., Hassoun J., Sun Y.-K. (2011). Energy Environ. Sci..

[cit3] Kim H.-S., Periasamy P., Moon S.-I. (2005). J. Power Sources.

[cit4] Christie A. M., Lilley S. J., Staunton E., Andreev Y. G., Bruce P. G. (2005). Nature.

[cit5] MacGlashan G. S., Andreev Y. G., Bruce P. G. (1999). Nature.

[cit6] Croce F., Appetecchi G. B., Persi L., Scrosati B. (1998). Nature.

[cit7] Zhang S. S. (2007). J. Power Sources.

[cit8] Zhang J., Chen S., Xie X., Kretschmer K., Huang X., Sun B., Wang G. (2014). J. Membr. Sci..

[cit9] Zhang J., Sun B., Huang X., Chen S., Wang G. (2014). Sci. Rep..

[cit10] Gadjourova Z., Andreev Y. G., Tunstall D. P., Bruce P. G. (2001). Nature.

[cit11] Raghavan P., Choi J. W., Ahn J. H., Cheruvally G., Chauhan G. S., Ahn H. J., Nah C. (2008). J. Power Sources.

[cit12] Raghavan P., Manuel J., Zhao X., Kim D.-S., Ahn J.-H., Nah C. (2011). J. Power Sources.

[cit13] Song X., Ding W., Cheng B., Xing J. (2017). Polym. Compos..

[cit14] Saufi S. M., Ismail A. F. (2002). Songklanakarin J. Sci. Technol..

[cit15] Hwang Y. J., Nahm K. S., Kumar T. P., Stephan A. M. (2008). J. Membr. Sci..

[cit16] Sohn J. Y., Im J. S., Gwon S. J., Choi J. H., Shin J., Nho Y. C. (2009). Radiat. Phys. Chem..

[cit17] Puguan J. M. C., Chung W.-J., Kim H. (2016). Electrochim. Acta.

[cit18] Kim K. M., Park N.-G., Ryu K. S., Chang S. H. (2006). Electrochim. Acta.

[cit19] Aravindan V., Vickraman P. (2008). J. Appl. Polym. Sci..

[cit20] Choi Y., Zhang K., Chung K. Y., Wang D. H., Park J. H. (2016). RSC Adv..

[cit21] Akin I., Zor E., Bingol H., Ersoz M. (2014). J. Phys. Chem. B.

[cit22] Lee J. K., Nath N. C. D., Cha E. H., Sarker S., Park H. S., Jeong W. S., Hong S. H., Lee J. J. (2010). Bull. Korean Chem. Soc..

[cit23] Farooqui U. R., Ahmad A. L., Hamid N. A. (2018). Renewable Sustainable Energy Rev..

[cit24] Abbrent S., Plestil J., Hlavata D., Lindgren J., Tegenfeldt J., Wendsjö Å. (2001). Polymer.

[cit25] Chen D., Tang L., Li J. (2010). Chem. Soc. Rev..

[cit26] Kalyana Sundaram N. T., Subramania A. (2007). Electrochim. Acta.

[cit27] Moreno M., Quijada R., Santa Ana M. A., Benavente E., Gomez-Romero P., González G. (2011). Electrochim. Acta.

[cit28] Costa C. M., Silva M. M., Lanceros-Méndez S. (2013). RSC Adv..

[cit29] Deimede V., Elmasides C. (2015). Energy Technol..

[cit30] dong Kang G., ming Cao Y. (2014). J. Membr. Sci..

[cit31] Bhadra S., Khastgir D., Singha N. K., Lee J. H. (2009). Prog. Polym. Sci..

[cit32] Zheng J., Ma X., He X., Gao M., Li G. (2012). Procedia Eng..

[cit33] Mooss V. A., Athawale A. A. (2016). J. Polym. Sci., Part A: Polym. Chem..

[cit34] Nabi S. A., Shahadat M., Bushra R., Oves M., Ahmed F. (2011). Chem. Eng. J..

[cit35] Farooqui U. R., Ahmad A. L., Hamid N. A. (2017). Polym. Test..

[cit36] Konios D., Stylianakis M. M., Stratakis E., Kymakis E. (2014). J. Colloid Interface Sci..

[cit37] Marjanović B., Juranić I., Mentus S., Ćirić-Marjanović G., Holler P. (2010). Chem. Pap..

[cit38] Sudesh, Kumar N., Das S., Bernhard C., Varma G. D. Supercond. Sci. Technol..

[cit39] Vargas L. R., Poli A. K., Dutra R. d. C. L., de Souza C. B., Baldan M. R., Gonçalves E. S. (2017). J. Aerosp. Technol. Manage..

[cit40] Ahmad A. L., Farooqui U. R., Hamid N. A. (2018). Polymer.

[cit41] Masoud E. M. (2016). Polym. Test..

[cit42] Ulaganathan M., Mathew C. M., Rajendran S. (2013). Electrochim. Acta.

[cit43] Arora P., Zhang Z. (2004). Chem. Rev..

[cit44] Liu J., Conry T. E., Song X., Doeff M. M., Richardson T. J. (2011). Energy Environ. Sci..

[cit45] Yamada A., Koizumi H., Nishimura S., Sonoyama N., Kanno R., Yonemura M., Nakamura T., Kobayashi Y. (2006). Nat. Mater..

